# Machine learning utilising spectral derivative data improves cellular health classification through hyperspectral infra-red spectroscopy

**DOI:** 10.1371/journal.pone.0238647

**Published:** 2020-09-15

**Authors:** Ben O. L. Mellors, Abigail M. Spear, Christopher R. Howle, Kelly Curtis, Sara Macildowie, Hamid Dehghani

**Affiliations:** 1 Physical Sciences for Health Centre for Doctoral Training, College of Engineering and Physical Sciences, University of Birmingham, Birmingham, United Kingdom; 2 School of Computer Science, College of Engineering and Physical Sciences, University of Birmingham, Birmingham, United Kingdom; 3 Defence Science and Technology Laboratory, Porton Down, Salisbury, United Kingdom; Tianjin University, CHINA

## Abstract

The objective differentiation of facets of cellular metabolism is important for several clinical applications, including accurate definition of tumour boundaries and targeted wound debridement. To this end, spectral biomarkers to differentiate live and necrotic/apoptotic cells have been defined using *in vitro* methods. The delineation of different cellular states using spectroscopic methods is difficult due to the complex nature of these biological processes. Sophisticated, objective classification methods will therefore be important for such differentiation. In this study, spectral data from healthy/traumatised cell samples using hyperspectral imaging between 2500–3500 nm were collected using a portable prototype device. Machine learning algorithms, in the form of clustering, have been performed on a variety of pre-processing data types including ‘raw’ unprocessed, smoothed resampling, background subtracted and spectral derivative. The resulting clusters were utilised as a diagnostic tool for the assessment of cellular health and quantified using both sensitivity and specificity to compare the different analysis methods. The raw data exhibited differences for one of the three different trauma types applied, although unable to accurately cluster all the traumatised samples due to signal contamination from the chemical insult. The background subtracted and smoothed data sets reduced the accuracy further, due to the apparent removal of key spectral features which exhibit cellular health. However, the spectral derivative data-types significantly improved the accuracy of clustering compared to other data types, with both sensitivity and specificity for the background subtracted data set being >94% highlighting its utility to account for unknown signal contamination while maintaining important cellular spectral features.

## Introduction

Infrared (IR) hyperspectral imaging and spectroscopy methods have been used widely in clinical applications for a variety of medical problems since the 1990s [1]. One of the most common areas for this technology is within the field of wound healing and diagnostics, covering a range of medical applications including diabetic foot ulcers [2] and burns [3]. These methods detect spectral information from the underlying biology and assess differences between healthy and non-healthy tissue and their cellular constituents. Despite these advances, many of such methods are still focused upon the Near Infrared (NIR) optical window, while there has been some insight into the short-wave (SWIR) region, incorporating 900 to 2500 nm [4], where complementary information can assist any assessment or diagnosis. This has also been extended further into the mid-wave (MWIR) region with both IR and Raman microspectroscopy [5], but little work has been done using macro tissue or cellular models.

Spectroscopic imaging in the extended IR region, beyond the conventional NIR methods used, requires additional considerations for both sample preparation and imaging methodology. Due to the high absorption of water within the SWIR/MWIR region, biological samples are often chemically ‘fixed’ to remove the unwanted water signature [6], however this process can also remove significant spectral features for accurate classification [7]. Spectroscopic images of these fixed samples are collected using Atomic Force Microscopy (AFM), requiring the sample to be placed in a vacuum, further reducing the availability of imaging within this region in most studies. Despite these challenges, imaging further into the IR window could potentially provide complementary information about specific spectral features such as lipids, collagen and other cellular constituents for clinical diagnostics, alongside current imaging modalities including spatial frequency domain imaging (SFDI) [8], laser doppler perfusion imaging (LDPI) [9] and thermography [10], which are more readily available.

Most methods used to date investigate *ex vivo* tissue samples only, which contain multiple signals from bulk tissue, such as cells, blood, and other tissue constituents. These mixed signals are difficult to separate, prompting work towards *in vivo* methods of individual elements contributing to detectable spectral features. Live cell imaging methods have been advancing in the last two decades, through the use of microspectroscopy and Raman spectroscopy [11, 12]. Initially these required the use of high-power IR sources, such as synchrotron beams, to resolve the spectra from cellular constituents. These also often require specialised cell preparations, including fixing and drying of samples, which have been shown to exhibit a loss in cellular content, effecting the resulting IR spectra and detectable contrast [13]. More recently, the work has moved towards custom imaging systems for live cell analysis, such as the use of narrow viewing windows and IR transparent housing [14]. It has been shown that in both the SWIR and MWIR, spectral regions of interest corresponding to lipids (1200, 1400, 1700, and 3333–3533 nm), collagen (1200 and 1500 nm) and other cellular constituents are detectable [12, 15, 16]. These studies highlight the need for further inspection into the IR region, with the need to target several different wavelengths of interest across a broad spectral range using a hyperspectral approach that will maximise signal contrast and features, while minimizing signal contamination from its local sampling environment.

Compared to the methods discussed, hyperspectral methods utilise up to three orders of magnitude more wavelengths, vastly increasing the size and dimensionality of the resulting data-set. This subsequently creates an additional need for intelligent data analysis to produce both reliable and easy-to-interpret results for the clinical setting. In traditional clinical imaging, simple statistics such as mean values, standard deviation and range, of simple detectable characteristics such as intensity of each pixel within a single image, or the differences across a temporal data-set are used to aid clinical diagnosis. However, for larger dimensional data-sets this provides a challenge with the increased number of variables and dependability of these values across samples, imaging environment and sample preparation, i.e. signal contamination. The use of machine learning (ML) in medical applications is not new [17], although the majority of these methods use supervised learning approaches. These methods require large data-sets to allow a suitable number for training/testing, which are not often available within pre-clinical studies, where they are developed and implemented. Unsupervised methods, such as clustering, alongside dimensionality reduction methods, offers an alternative which has been implemented in this work. Although it can be argued that unsupervised learning would in practice requires a much larger set of data, it has been applied to other works [18] and has been implemented for this preliminary study. Live cell hyperspectral imaging for assessing wound healing, with the use of machine learning, has been previously investigated within the visible-NIR range. 3D-cell cultures were grown, and trauma induced via a biopsy punch method, with clustering methods applied as no a-priori information for each class was available [19].

k-means clustering provides a method in which a pre-defined number of clusters can be used. For a binary diagnostic problem of healthy versus death as used for this study, k = 2, where k is the number of required classes or clusters, creating a two-cluster problem designed for the separation of the two different cell states. k-means is often used in medical image processing to provide an improvement in image segmentation of different tissue types or classes, where ML tool is specifically applied to increase the accuracy of segmentation, as well as the total throughput of images as compared to human image analysis [20]. k-means has also been applied in the field of spectral analysis for clinical diagnosis, with endoscopic imaging producing a large spectral data-set, which were sampled and analysed using a pre-determined number of clusters to represent the different clinical diagnoses [21]. These applications, although just an example and not completely covering the vast amount of work undertaken in this field, highlight the ability of k-means clustering as an intuitive, controllable, and simple method for grouping similar data for diagnostic purposes.

Principal Component Analysis (PCA) is an additional tool used for high dimensional data to aid in the clustering/classification process. PCA reduces the dimensionality of the data by re-representing the information onto a set of principal components that highlight the largest variability within the data-set [22]. PCA is often used in Raman spectroscopy applications, for example, where spectral information is collected from different biological systems, such as single cells or tissue samples, with many high dimensional spectra collected from each system. PCA aids in the extraction of the differences between the obtained spectra, whilst reducing the dimensionality, aiding further with the post-processing computational time [23] and is commonly applied in spectral analysis prior to k-means clustering [24], whilst it has also been previously applied to hyperspectral imaging using additional clustering methods [25].

All the classification methods outlined above utilise data from an imaging modality which typically undergoes pre-processing to improve classification output, such as background noise removal, spectra/temporal smoothing, bias removal, and averaging. Such data pre-processing converts raw measured data to different ‘data-types’ typically containing different information content. The use and effect of such data-types and utilisation of any classification algorithm, then itself becomes an important issue to better understand the information content and feature selection for classification.

Here a combination of the use of IR hyperspectral imaging within the SWIR/MWIR window is presented alongside the use of unsupervised machine learning methods for diagnostics analysis. The combination of these two techniques, which combines the imaging method’s ability to detect subtle spectral differences between cell culture models and the ability of ML to identify and classify these, produces a novel approach for the detection and diagnosis of cellular health. A live cell model was created, containing cells of relevance to wound biology and with facets that permitted interrogation in the mid-IR range and cell death induced. Spectral data was collected from live and dead/dying cells with the aim of determining spectral differences and grouping the two different cell states. A total of 72 samples were collected, with an equal split between healthy and treated cell cultures. k-means clustering and PCA were applied to different data-types, to determine which method provides the most accurate and reliable diagnostic tool. The aim of this work is to provide a binary classification tool for the diagnosis of the state of cell health. The sensitivity and specificity of the obtained clusters for each data-type and associated algorithm is reported to allow for a quantifiable comparison of the diagnostics tool’s ability to accurately diagnose the two different cellular health states.

## Materials and methods

### Cell culture

Human dermal fibroblast (HDFa, Gibco) cells were cultured in 12-well Costar Transwell inserts with a polyethylene terephthalate (PET) membrane (Corning) in Medium 106 supplemented with Low serum growth supplement (all Gibco) at liquid-liquid interface (LLI) (4 inserts/plate). Growth medium was removed from inside the insert and reduced to 0.5 mL below the insert to achieve air-liquid interface (ALI), immediately before infrared imaging.

Conditions for inducing necrosis and apoptosis were derived from the literature and confirmed for this cell line [26, 27]. Cells were seeded at 1 x 10^5^ cells/cm^2^ and incubated at 37°C, 5% CO_2_ overnight for at least 90% confluency the following morning. Necrosis was induced using 0.01% (v/v) Triton X-100 (TX100, Sigma) for 1 hr in serum free media (SFM) or 5 mM H_2_O_2_ in DMEM (Sigma) for 4 hr. Apoptosis was induced using 100 μM H_2_O_2_ for 4 hr. Cell death was confirmed by staining with Calcein AM (Invitrogen) and propidium iodide (Sigma), or Apoptosis/Necrosis Detection Kit (Abcam).

For each experimental data set collected (biological replicate), two technical replicates were completed each for untreated, healthy controls, and treated samples. This imaging set-up was repeated for each of the different trauma types applied. A total of 18 repeats were collected across the three different trauma protocols, creating 72 (18 x 4) insert measurements of 36 healthy and 36 traumatised cell cultures.

### Cell staining

To confirm the apoptosis or necrosis of the cells following treatment, cells were stained with culture medium containing 2ug/ml Calcein AM and 2 μg/ml propidium Iodide, or the Apoptosis/Necrosis Detection Kit according to manufacturer’s protocol. Cells were visualised using an ImageXpress Pico cell imager (Molecular Devices).

**Fig 1:**
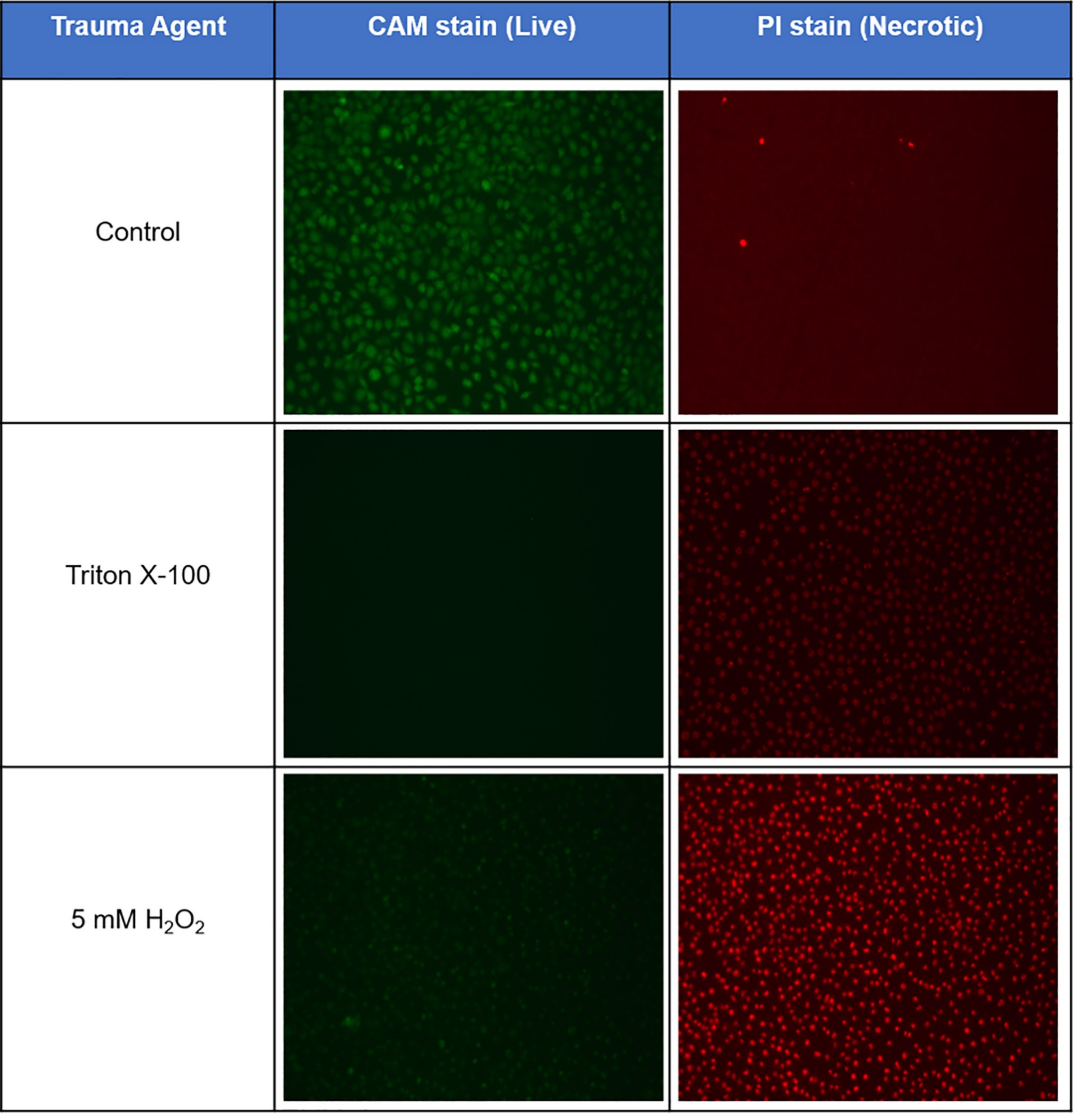
Necrotic cell staining. Confirmation of cellular trauma was achieved through the staining of treated cells. Necrotic staining was achieved using an Apoptosis/Necrosis Detection Kit (Abcam), with CAM stain (green) and PI stain (red) for the live/necrotic cells, respectively. These staining images show both the Triton X-100 and higher concentration of H_2_O_2_ produced >99% necrosis within the culture.

Fig 1 shows an example of the staining images collected for both the Triton X-100 and 5 mM H_2_O_2_ Apoptosis/Necrosis Detection Kit. The green CAM stain highlights the live cells, while the red PI stain the necrotic. Theses stains, along with those for apoptosis, confirmed the correct levels of chemical trauma were applied for the desired cellular health outcome. This further highlights the importance of an objective method for classification, with only estimates of the quantification of cellular death possible, along with the importance of non-invasive imaging methods suitable for *in vivo* techniques, in contrast to the cell straining utilised here.

### Infrared imager

Hyperspectral images of the 12-well plates for the trauma study were collected using a prototype negative contrast imaging device (NCI) [28]. This NCI device, developed by M Squared Lasers (Glasgow, UK), is a reflectance imaging device that collects hyperspectral images from illumination in both the short and mid-wave infrared regions (SWIR/MWIR) and has been used to identify spectral differences between wound biopsies, with seven human samples being imaged to predict their wound healing outcome [29].

**Fig 2:**
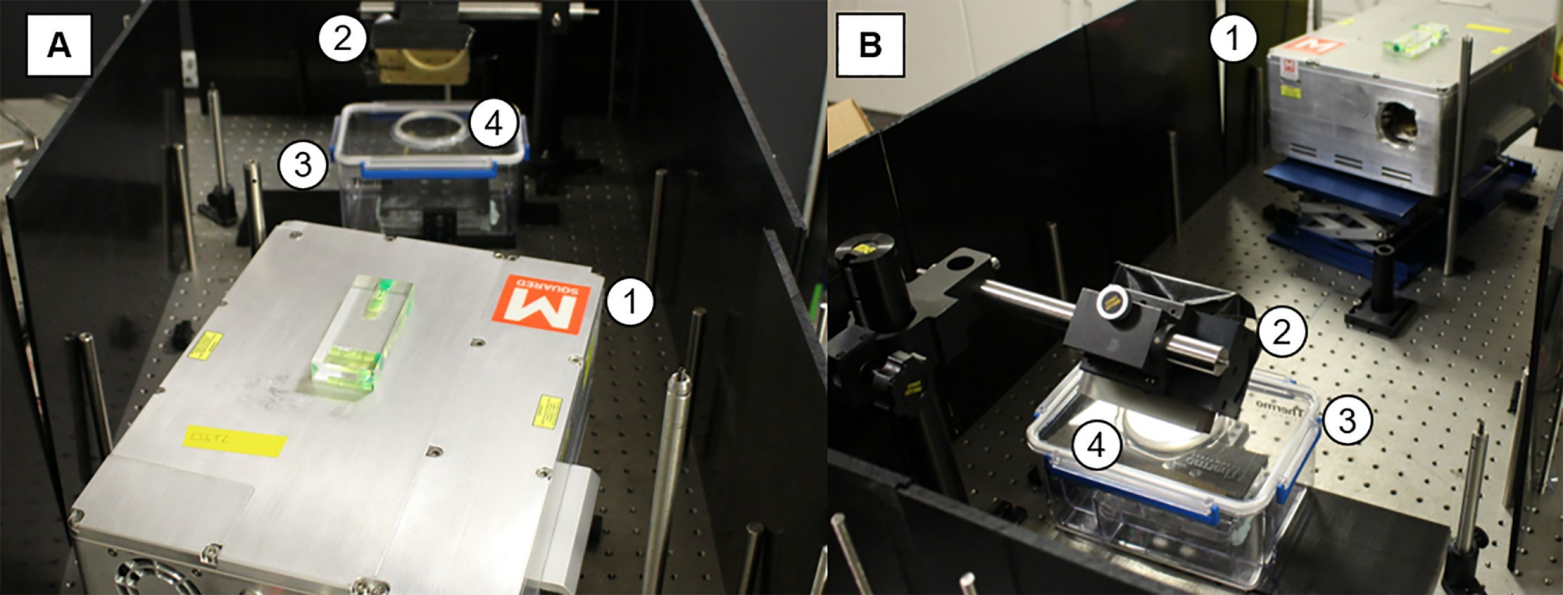
Negative Contrast Imaging (NCI) system set up. Images A and B show the set up used for collecting the hyperspectral images of the 12-well plates containing the cellular inserts. (1) Main NCI device (2) Gold coated steering mirror (3) Custom built imaging box (4) Calcium fluoride window.

Fig 2 shows the system set-up for imaging of the cell inserts. The NCI device (1) outputs light at discrete wavelengths which then propagates to a gold coated steering mirror (2). This mirror reflects the light into the custom-built imaging housing (3), which maintains the sterile environment for the cell culture. The housing has a built-in calcium fluoride (CaF_2_) viewing window (4), which is transparent within the wavelength region used for this study. Preliminary testing of reflectance phantoms within the custom-built housing was performed to assess the effects of any stray light and ensure the correct alignment of the gold-coated steering mirror and CaF_2_ viewing window.

**Fig 3:**
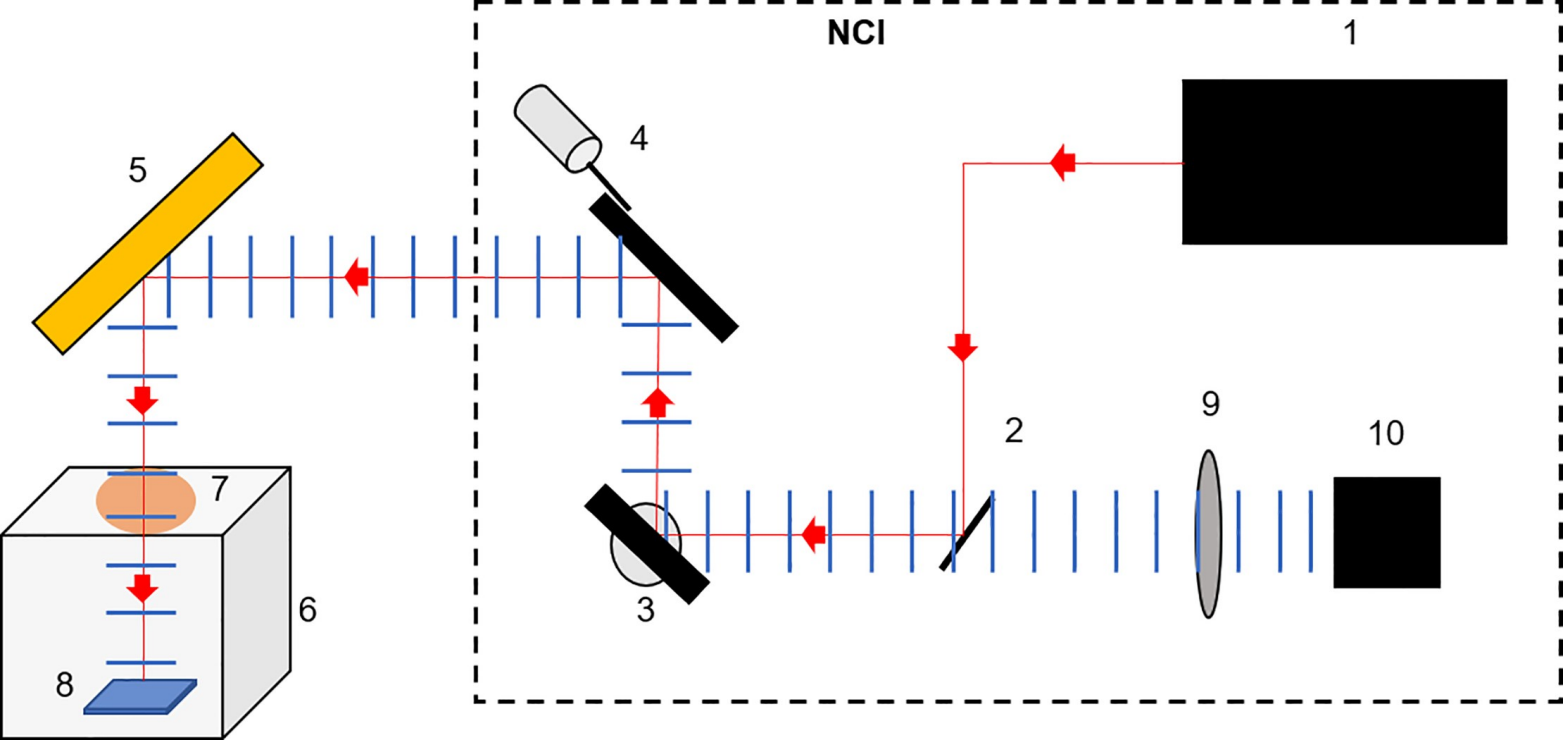
Schematic representation of the imaging set up. (1) The Intracavity Optical Parametric Oscillator (ICOPO) provides the IR illumination for measurements. The (2) gold coated steering mirror, (3) y-axis galvanometer mirror and (4) x-axis galvanometer mirror are also housed in the NCI system. (5) External gold coated steering mirror, (6) custom built transport chamber, (7) Calcium Fluoride (CaF_2_) viewing window all allow for the imaging of the (8) sample, within a sterile environment. The reflected light then retraces its path before passing through a (9) CaF_2_ focusing lens and onto the (10) MCT detector.

Fig 3 shows a schematic representation of the same set up, including the inner workings of the NCI, used to collect the hyperspectral data for each sample. All elements housed within the NCI device are contained by the dashed box, and a full technical description has been previously published [29]. The Intracavity Optical Parametric Oscillator (ICOPO) provides with illumination in the MWIR range used for this study (1). The outgoing beam is directed using an internal gold coated steering mirror (2) towards the two galvanometer mirrors controlling the y-axis (3) and x-axis (4) before leaving the NCI housing. The beam then propagates towards the external gold coated steering mirror (5) which directs the beam vertically towards the sample. The custom-built sterile chamber (6) contains a CaF_2_ IR transparent optical window (7) allowing the transmission of the beam to the sample (8). The reflected light then travels back to the NCI system via the external steering mirror (5) and is directed towards the detection optics with the two galvanometers (4) and (3) internally. A CaF_2_ focusing lens (9) is then used before the reflectance signal is collected by a Zn doped MCT detector (10).

Images were collected using the NCIs built-in spectroscopy mode. The image resolution was selected at the highest possible value, 512 × 512 pixels, with the largest system magnification generating a field of view of 550 × 550 mm, resulting in a pixel size of 0.93 mm. Within imaging mode, the range and spectral resolution can be controlled, with 2500–3500 nm at 10 nm chosen respectively giving a total of 101 wavelengths. These settings, along with the raster scan speed of the NCI, resulted in a total imaging time of <7 seconds per image, including data transfer. These spectral images were then combined to produce a 3D data hypercube for analysis.

### Image analysis

**Fig 4:**
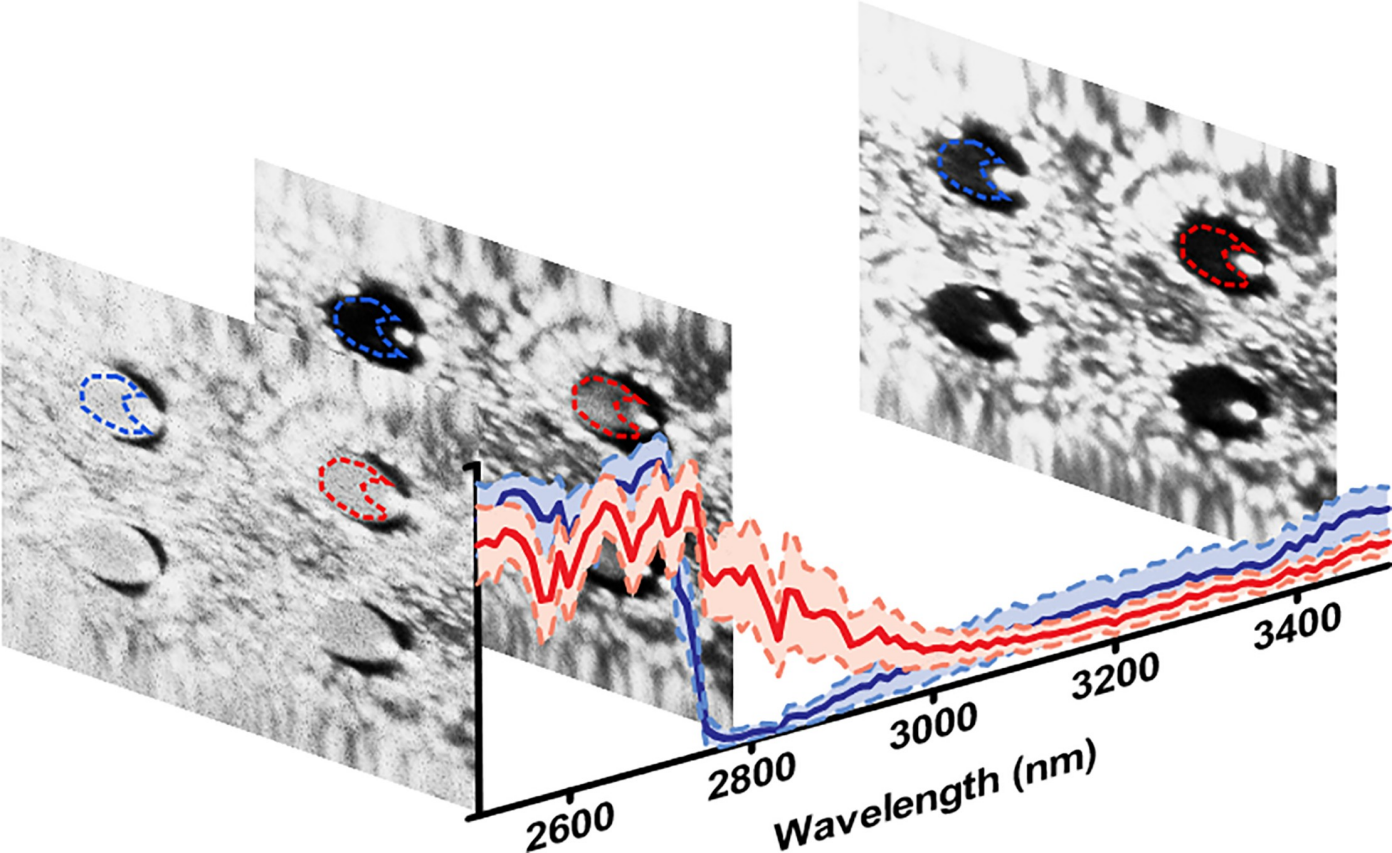
3D hyperspectral data cube. Visual representation of three example images from the NCI imager forming a 3D hypercube. The corresponding ‘Raw’ spectra of the mean of each ROI is also shown. The blue spectrum represents a healthy sample, and the red a traumatised sample treated with Triton X. A region of interest was obtained for each insert to collect the spectra from each sample, removing the spectral contribution of the internal reflection saturating the detector. This can be observed in the right-hand side of each well within the images.

Image analysis and the resulting ML applications were all performed using MATLAB® (MATHWORKS). The data was grouped to form a 3D hypercube with x and y dimensions of each image corresponding to the first two dimensions (512 × 512), and the wavelength corresponding to the third (101). Each 3D data hypercube (512 × 512 × 101) was analysed individually to extract the spectrum for each of the 4 different inserts in the NCI field-of-view. Example images are shown in Fig 4 as taken at 2500, 2800 and 3450 nm to highlight the differences between the healthy (Blue) and traumatised (Red) ‘Raw’ spectra, with the standard deviation shown, representing a sample from the 101 images which make up the hypercube for each sample. For each insert, a region of interest (ROI) based on a-priori knowledge for the location of the well was drawn with specific attention to ensure exclusion of the region of internal reflection from the source within each well and contains all pixels within and on the dashed lines. This can be observed in the images as the area of saturation in the right-hand side of each of the 4 inserts, creating a ‘C’ shape area for each ROI. This region was first outlined using the 2500 nm image to identify the bounds of the cell inserts, which was clearly visible at this wavelength. The exclusion region was then identified using images at the higher wavelengths and systematically checked at different wavelengths to ensure no internal reflection is part of each insert ROI.

These ROIs are then propagated throughout the hypercube for each data set, to resample the full spectra from each insert, within all 18 repeat measurements for the three different trauma protocols, which were then analysed by combining the 72 (18 × 4 inserts) individual ROI spectra to generate the large data-set containing all the combined 36 healthy and 36 traumatised samples.

### Machine learning for cellular health classification

Once the spectra for each sample had been obtained, the next step was to assess different pre and post processing methods for the measured and re-sampled raw data. A variety of different methods were applied, which are next outlined.

#### Data Pre-processing

Three different pre-processing methods have been used in this study, which were combined to generate 4 additional data-types, alongside the conventionally used raw ROI mean. The first involved utilisation of the ‘smoothts’ function within MATLAB to smooth the spectra for each data set. This function was chosen due to its ability to control the size and characteristics of the smoothing function, as compared to other available smoothing functions. A 5-point window size was used along with a gaussian smoothing function with a 0.5 standard deviation, which was applied to each spectrum from the ROI of the corresponding insert. Each smooth spectral point was calculated as follows,

$$R_{\left(\lambda \right)}=\sum_{\lambda -2}^{\lambda +2}R_{\left(\lambda \right)}∙\hat{G},$$ (1)

where, *R* is the raw reflectance data at wavelength *λ* and *Ĝ* is the normalised gaussian function in 1D, with a window size 5, standard deviation of 0.5, as implemented within the ‘smoothts’ MATLAB function. These window parameters were chosen to reduce the noise within each spectra, whilst maintaining the dominant spectral features.

**Fig 5:**
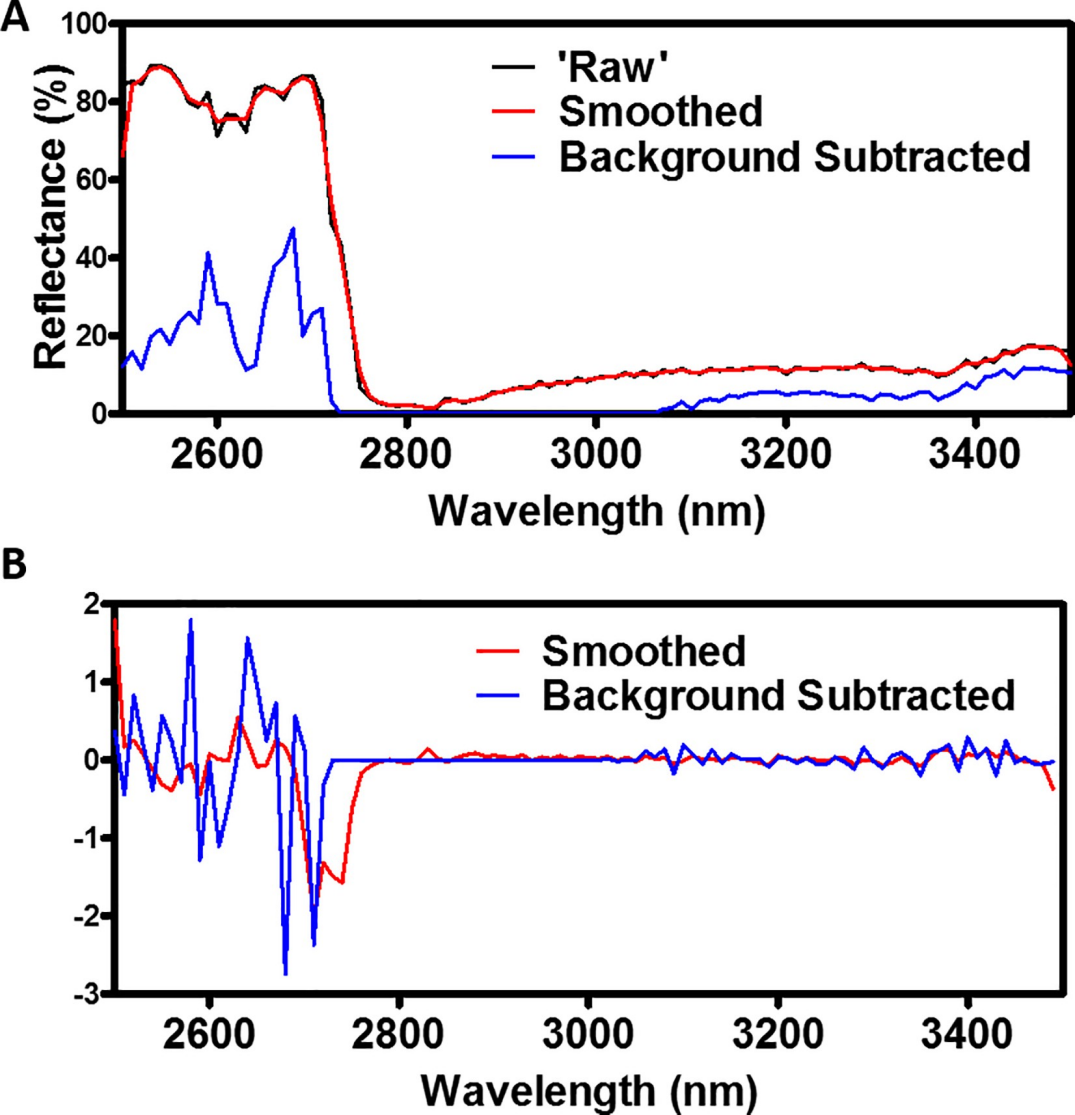
Pre-Processing Data-type visualisations. A: Spectral comparison between the ‘Raw’, smoothed and background subtracted data-types for a single traumatised spectrum. B: Spectral derivative comparison for both the smooth and background subtracted data sets shown in A.

The second method applied was a simple background correction, which allows for the consideration of any spectral contributions from both the cell trauma method as well as the cell inserts. A supplementary data set was also collected for the three different trauma types and healthy controls. For each of these, the treatment media was applied to the standard four inserts set up, with no cells seeded. The same spectra, using the NCI, were collected and the spectra of each of the four inserts were then averaged (mean) to generate the background spectra for subtraction. Each spectra from the inserts containing live cells were then simply matched with the corresponding media background spectra, which was then subtracted for each wavelength to generate the background (BG) subtracted data-type.

$$R_{BG(\lambda )}=R_{\left(\lambda \right)}-M_{\left(\lambda \right)},$$ (2)

where, *R* is the raw reflectance data and *M* is the reflectance data from the corresponding media only spectrum from the same trauma method at wavelength *λ*. As the cell insert preparation protocol is independent of the trauma method, removal of the background will highlight the differences between the healthy and traumatised sample spectra only and should remove any contributions from the cell trauma method or cell culture plastics/insert. Each of these three data-types are shown in Fig 5A for comparison, showing a single traumatised spectrum.

The final pre-processing applied was to calculate the spectral derivate of each data set. Derivative spectroscopy methods have been used since the 1950s, becoming more common place in the 1970s with the increased computational power available at the time. These methods are now common places in a variety of disciplines to eliminate any background signals and for resolving overlapping spectral features [30]. While these methods can be shown to amplify noise, derivative-based methods consider the direct relationship between the nearest neighbour wavelength measurements and hence can account for any spectrally independent systematic noise [31, 32]. This includes the field of wound healing, where derivative spectral methods have been utilised in Fourier Transform Infrared Spectroscopy for the classification of the spectral patterns for burn wound healing [33]. The spectral derivative, *R*_*SD*_ can be calculated as show in Eq 3 below,

$$R_{SD(\lambda )}=\frac{dR}{d\lambda }=\frac{R_{\left(\lambda +1\right)}-R_{\left(\lambda \right)}}{(\lambda +1)-\lambda },$$ (3)

where *R*_*λ*_ is the reflectance spectral value at a given wavelength *λ*. This method was applied to both the smoothed and BG subtracted data, generating the 4 additional data-types mentioned, which along with the raw ROI mean data, make up all the data-types tested for this study. An example of the 1^st^ derivative of the smoothed and background subtracted spectra example are shown in Fig 5B.

#### Post processing

Each of the five data-types outlined above, as shown in Table 1, were also subjected to two different post processing steps, clustering, and PCA-clustering. In this study, the popular k-means clustering algorithm, first described in the late 1960s [34], has been used to separate the data into ‘healthy’ and ‘traumatised’ clusters, which was then compared to the ground truth labels. k-means clustering is a form of unsupervised learning in which a set of data points are assigned to a cluster using a proximity metric [35]. For this study, two clusters (k = 2) were considered to represent the ‘healthy’ and ‘traumatised’ groups. The algorithm works by assigning k number of clustering centres randomly in an N-dimensional space, where N is the dimensionality of the spectral data. The proximity metric, with cosine distance being used in this work, is then utilised to assign each point of the data set to its nearest clustering centre. The clustering centre is then re-calculated based on the mean N-dimensional co-ordinates of its data point members before the process of data point assignment is repeated. These steps repeat until a convergence is reached, where no single data point changes its cluster label following the previous re-calculation.

**Table 1:** Clustering frequency results for each of the pre- and post-processing data-type.

Pre-Processing Data-type	Post-Processing Data-type	
	k-means	PCA and k-means
‘Raw’	98.5	98.2
Smoothed	93.0	99.3
Background Subtracted	100.0	100
Spectral Derivative of Smoothed	47.6	34.7
Spectral Derivative of Background Subtracted	52.1	39.6

The k-means clustering was applied to each of the five different pre-processed data-types to generate the clustering labels needed for their comparison. Due to the initial random assignment of the initial k cluster centres, the results can be subject to variation. Therefore, the k-means process was repeated 5000 times, producing a ≤1% variation in the clustering frequency, which was then defined as the number of times the modal clustering arrangement was reached divided by the total number of k-means repeats.

**Fig 6:**
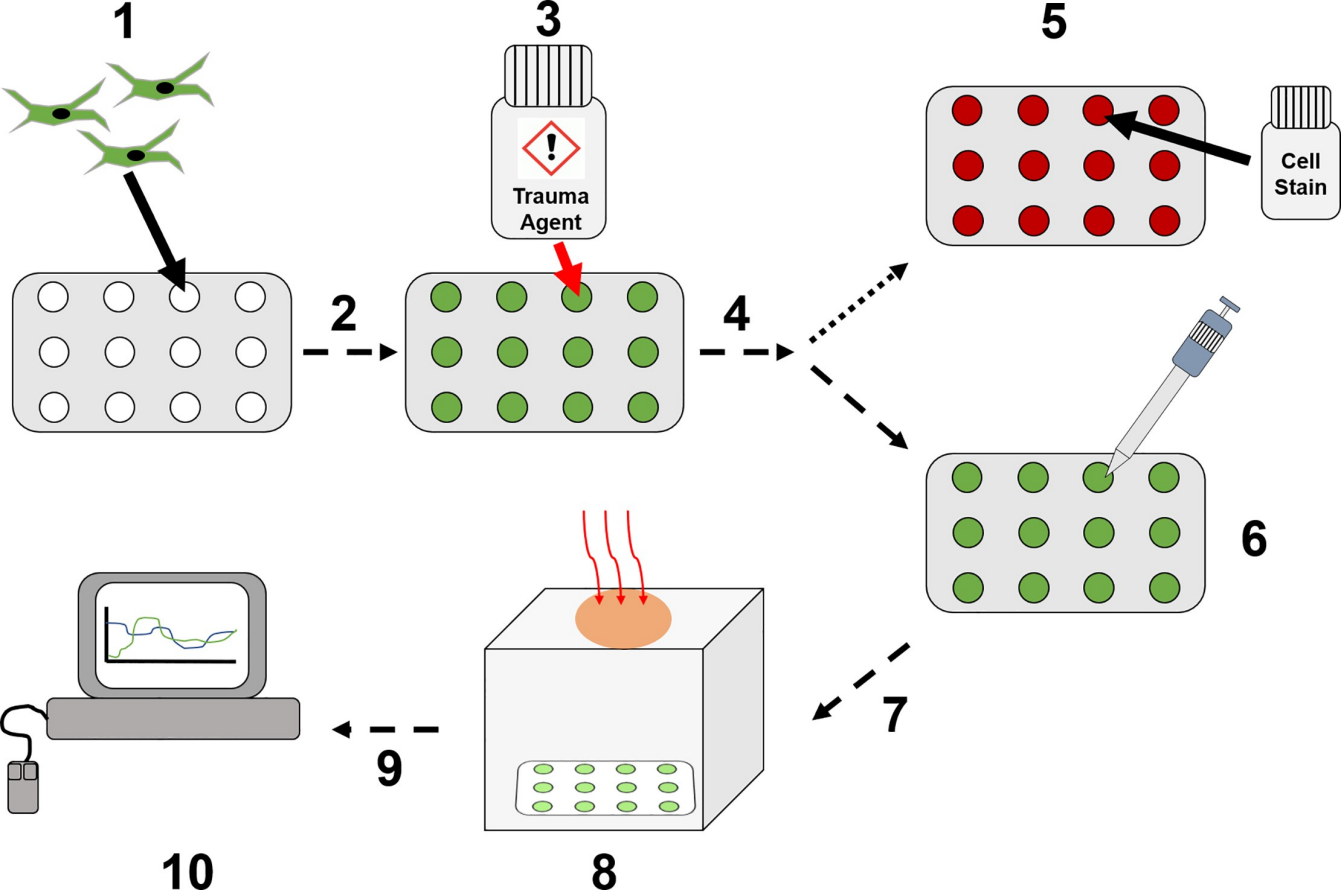
Data collection workflow. (1) HDFa cells are seeded onto 12-well plate inserts. (2) Cultured overnight until >90% confluent. (3) Treated with cell trauma inducing agent. (4) Incubated for 1–4 hours dependent upon cell trauma agent. (5) Cell staining to confirm apoptosis/necrosis levels. (6) Reduction of growth medium to 0.5 mL for imaging preparation. (7) Transfer to imaging box for NCI data collection. (8) Image collection using NCI SWIR/MWIR detector. (9) Images analysed to produce cell spectral data. (10) Pre- and post-process spectral analysis.

The second and final post processing method applied was using PCA [36]. PCA is a common method applied to signal processing with a high dimensionality to the data set, reducing this dimensionality whilst maintaining the variance in the case of k-means clustering, a distance metric is used to measure the distance between points, which is used to determine which cluster any given point belongs to. This dimensional reduction technique was applied to each of the five different pre-processed data-types, reducing the original N-dimensional data set, N = 101, to that in which has M dimensions, representing ≥95% of the variance, such that M<<N. When the dimensionality of this data is high, these metrics can be affected by the sparsity of the data across all dimensions, resulting in poor clustering, an attribute of the ‘curse of dimensionality’. The use of PCA in wound healing hyperspectral imaging has been shown for the prediction of healing in diabetic foot ulcers, along with a threshold value for oxygenation or given principal components as a classification tool [37]. Research into clustering within high dimensional data has shown that, in general, reducing the number of dimensions improves the clustering using simple distance metrics, such as the cosine distance used in this study, but there is no general ‘one size fits all’ rule which can be applied to all data sets [38]. Therefore, both the full dimensional data sets, and those reduced by PCA were analysed to determine the optimal procedure to correctly cluster the two different cellular health states. A full breakdown on the data collection process is shown in Fig 6.

## Results

To compare the clustering results of each of the different data-types, two additional metrics were used alongside the clustering frequency. The sensitivity and specificity are defined as follows:

$$Sensitivty=\frac{\#True\;postives}{\#False\;Negatives+\#True\;Positives}$$ (4)

$$Specificty=\frac{\#True\;negatives}{\#True\;negatives+\#False\;positives}.$$ (5)

Here, a true positive result is one in which a traumatised sample is correctly labelled as ‘traumatised’ by the clustering algorithm, while a true negative is one where a healthy sample is labelled as ‘healthy’. These two metrics were chosen to provide additional information about the accuracy of the binary classification algorithm used within this study. An ideal medical diagnostic test will be 100% accurate, i.e. all healthy patients will be identified as healthy, and all diseased as diseased, with no incorrect diagnosis. An accuracy of 100% is highly unlikely, requiring careful consideration of any given tests reported accuracy value. This reported value is a combination of both the sensitivity and specificity and can therefore be used to hide a medical tests true ability to accurately diagnose all healthy and diseased patients. By considering the sensitivity and specificity of this diagnostic tool individually, a quantification for the number of false positives and negatives has been shown. This is important for this diagnostics tool, as the treatment, or lack of, to an incorrectly diagnosed diseased state can have detrimental effects on a patient’s outcome.

**Fig 7:**
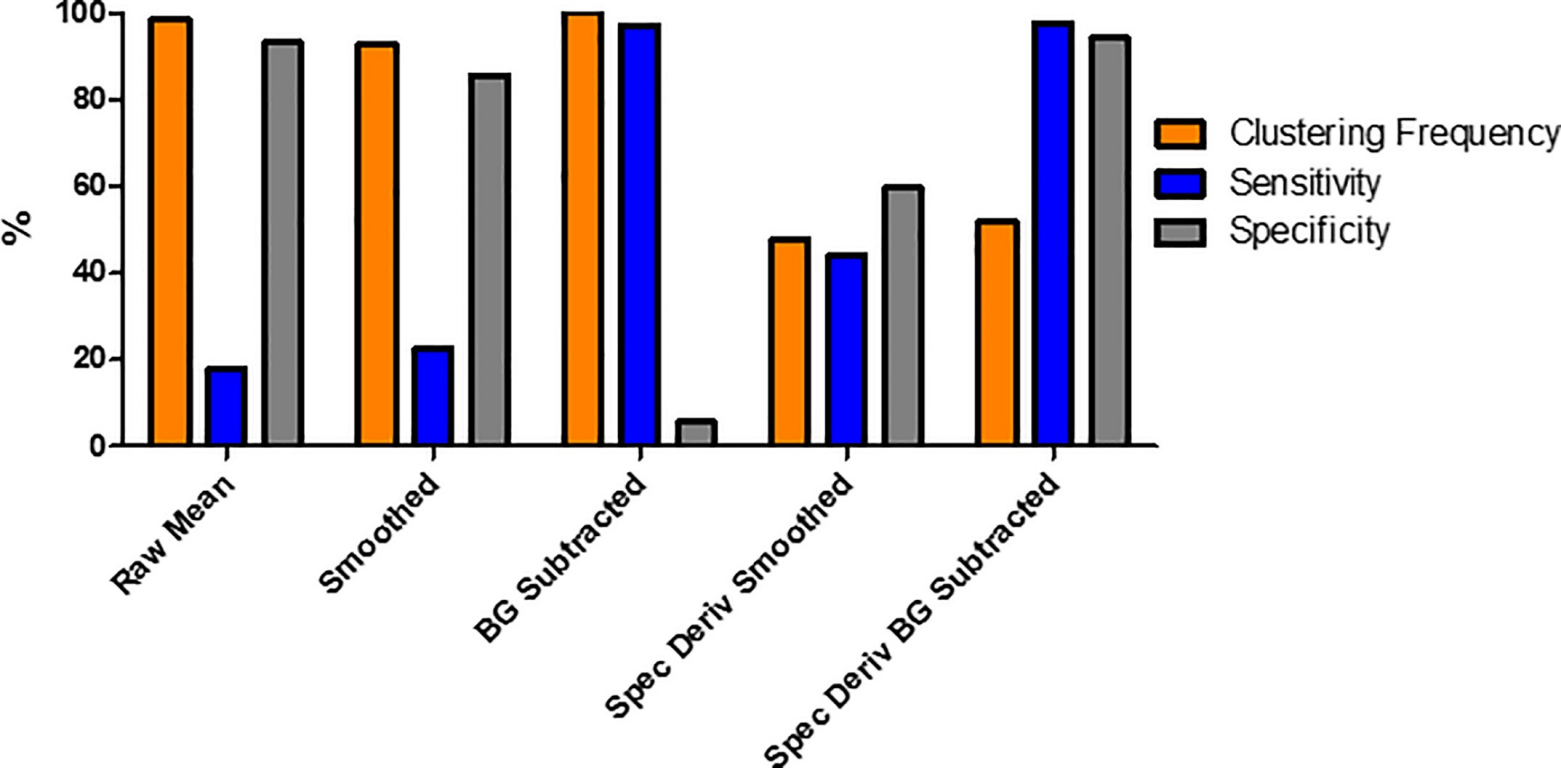
Data-type comparisons for non-Principal Component Analysis (PCA) data-types. Three different metrics, clustering frequency (Orange), sensitivity (Blue) and specificity (Grey) were used to quantitatively compare different data-types obtained from the raw hyperspectral images.

Fig 7 shows the results for each of the five different data-types being processed directly by the k-means algorithm. Here, the raw mean, smoothed and BG subtracted all exhibit clustering frequencies of >90%, however show low scores, <25%, for either sensitivity or specificity. The spectral derivate of the smoothed data showed an improvement upon the three previous data-types, with all three metrics above 40%, although the sensitivity is still below 50%. The most promising result comes for the spectral derivate of the BG subtracted data set. Here, both the sensitivity and specificity were >94%, while the clustering accuracy was above 50%.

**Fig 8:**
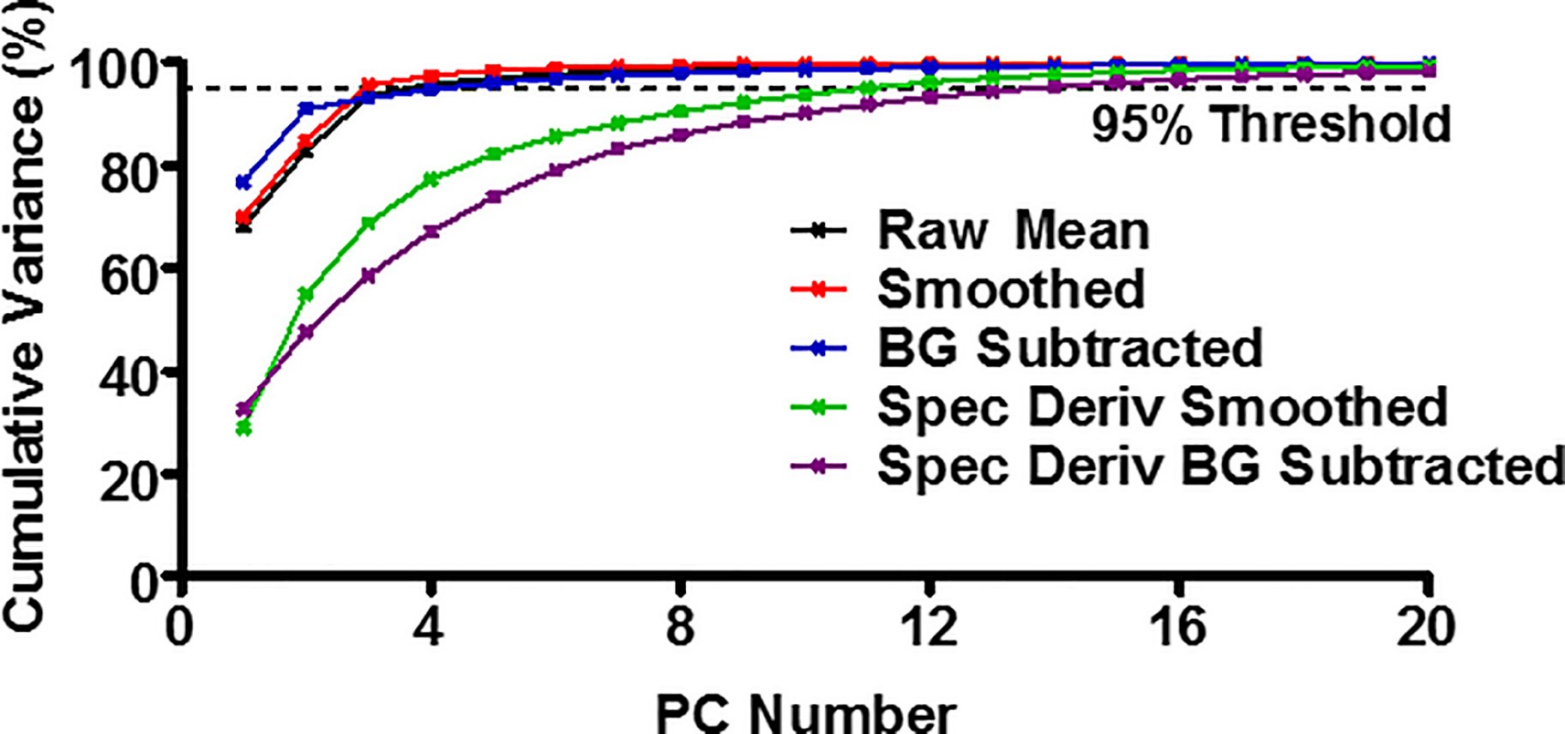
Principal Component Analysis (PCA) cumulative variance plots. For the five different pre-processing data-types, the plot of the principal components (PC) against the cumulative variance is shown. Each data-type was represented by the PC number which incorporated >95% of the variance in the PCA and k-means study.

This clustering was repeated for each of the five different data-types following the application of dimensional reduction, through the implementation of PCA. The number of principal components (PCs) for each data set was chosen such that >95% of the variance within the data set was represented, resulting in 4–14 PCs being considered, reducing the dimensionality from the original 101 dimensions, representing each of the wavelengths collected. The cumulative variance plots for each of the five different pre-processing types are shown in Fig 8, with the 95% threshold represented by a dashed line.

**Fig 9:**
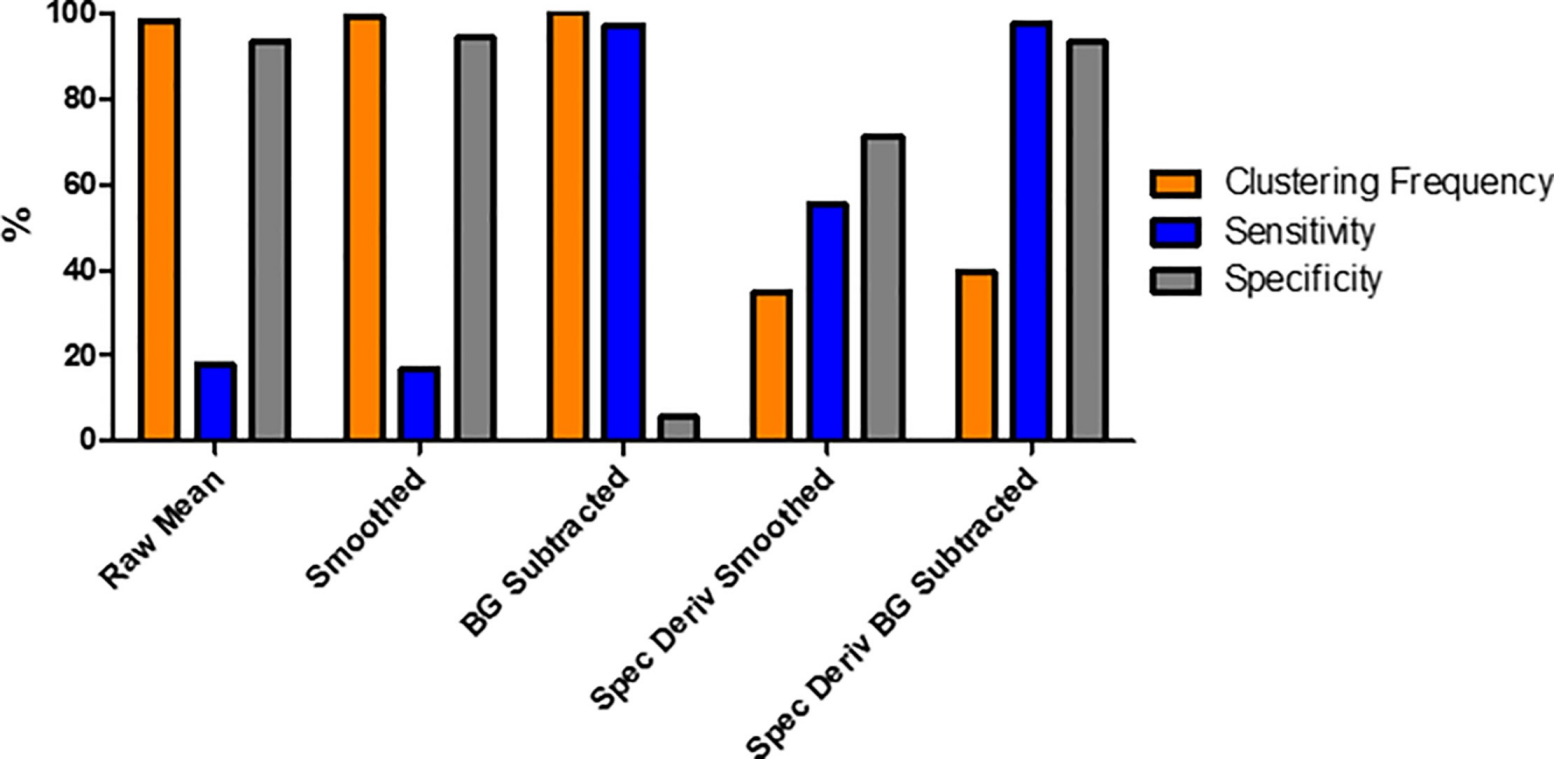
Data comparison for Principal Component Analysis (PCA) reduced data-types. Three different metrics, clustering frequency (Orange), sensitivity (Blue) and specificity (Grey) were used to quantitatively compare different data-types obtained from the raw hyperspectral images.

Fig 9 shows the k-means clustering quantification results for the PCA reduced data sets. Again, the five different data-types were compared using the clustering frequency, sensitivity, and specificity. Similar results were observed for the first three data-types; raw mean, smoothed and BG subtracted. These showed a high clustering frequency of >90%, along with a low, <20%, sensitivity and specificity. An improvement was seen with both the spectral derivative data-types. The spectral derivative of the smoothed data provided both a sensitivity and specificity of ~50%, while the clustering frequency was observed to be half of that seen in the non-PCA equivalent. As with the non-PCA result, the data set with the highest values for both sensitivity and specificity were found to be the spectral derivative of the BG subtracted data. Both values were >90%, although were slightly lower (0.3–1.1%) than the non-PCA data set, and the clustering accuracy was greatly reduced to <40%.

## Discussion

The spectral derivative of the background subtracted data has been shown to be an effective and reliable tool for diagnosing healthy and traumatised cellular samples. Any binary diagnostic test, such as one to define if a sample is healthy or not, can have its accuracy quantified using both sensitivity and specificity. For example, a test which has a low sensitivity and a high specificity, while accurate in labelling healthy samples correctly, the inability to identify traumatised samples would render the test unsuitable as a diagnostic tool. This outcome was observed in raw mean and smoothed data-types of the clustered (Fig 7) and PCA-clustered (Fig 9), with the full results shown within Tables 1–3 for the clustering frequency, sensitivity, and specificity respectively.

**Table 2:** Sensitivity results for each of the pre- and post-processing data-type.

Pre-Processing Data-type	Post-Processing Data-type	
	k-means	PCA and k-means
‘Raw’	17.8	17.8
Smoothed	22.5	16.9
Background Subtracted	97.2	97.2
Spectral Derivative of Smoothed	43.9	55.3
Spectral Derivative of Background Subtracted	97.8	97.6

**Table 3:** Specificity results for each of the pre- and post-processing data-type.

Pre-Processing Data-type	Post-Processing Data-type	
	k-means	PCA and k-means
‘Raw’	93.2	93.2
Smoothed	85.8	94.2
Background Subtracted	5.6	5.6
Spectral Derivative of Smoothed	59.7	71.3
Spectral Derivative of Background Subtracted	94.4	93.3

The opposite of this was seen in the background subtracted data, with high sensitivity but low specificity. While accurate at identifying those cells which are traumatised, it is poor at confirming cells which are healthy. In a clinical setting, this would translate to the potential treatment of healthy samples, wasting resources and time, or in wound debridement, to the unnecessary removal of healthy tissue which can cause additional problems in the wound healing process and in the patient’s future.

A significant improvement was seen in both spectral derivative data-types. The spectral derivative process adds to the complexity of the data by considering the changes in the reflectance spectra between neighbouring wavelengths. Through this simple step, additional bands in the data can be detected, and suitable spectral features enhanced. A previous study has looked at the effects of smoothing and spectral analysis up the exploration of subtle spectral differences [39]. However, the algorithms developed and optimised were modified for use with remote sensing data, therefore this study considers a more traditional spectroscopic method via hyperspectral image analysis. The outcomes of both this study and that of Tsai *et al* highlights the importance of additional post-processing spectral analysis to improve upon the identification of key spectral features [39].

The first spectral derivative data-type to discuss is the smoothed data. Although exhibiting a lower value for clustering frequency in both the non-PCA and PCA post-processing types, at ~50%, this is still a definitive clustering, with the 72 samples offering many possible clustering outcomes. Again, the sensitivity and specificity were around 50% for the two post-processing types. Despite this improvement, for a binary diagnostic test with an even distribution of healthy and traumatised samples, a 50% sensitivity and specificity would be achieved through a random assignment of ‘healthy’ and ‘traumatised’ clustering labels with a p = 0.5, q = 1-p probability respectively.

The final data-type tested was the spectral derivative of the background subtracted data. The raw background subtracted data exhibited a high clustering frequency and sensitivity, but the lowest specificity, ~6% for both post-processing types. Despite this, the spectral derivative of this data-type showed a significant improvement with the values for both sensitivity and specificity of >90% across all 5000 k-means clustering repeats. For the modal cluster, observed at a frequency of 52% and 38% for non-PCA and PCA respectively, all traumatised samples were correctly labelled while ≤3 of the healthy samples were incorrectly labelled as ‘traumatised’. In terms of a diagnostic test for wound healing, this method would provide an accurate and reliable measure for determining the status of cellular health.

For the data reduction method as applied in this study, PCA, the accuracy of the cell health clustering was comparable to the non-PCA data equivalents. Although the data dimensionality was reduced to as low as 3, compared to the full 101 wavelengths for the full data, different data reduction methods could also be investigated in future studies, with the aim of increased clustering accuracy and improved cluster grouping as represented by the silhouette score. Partial least squares (PLS) is an additional dimensionality reductions methodology that considers the correlation between both the dependant and independent variables, unlike PCA which only considers the independent variables [40]. Such methods have also been further developed with modified versions for analysis of Raman spectroscopy, with improved classification shown [41]. Dimensionality reduction could also be explored further through the use of autoencoders, which also consider non-linear contributions, although these require additional computation due to its neural network design [42].

**Fig 10:**
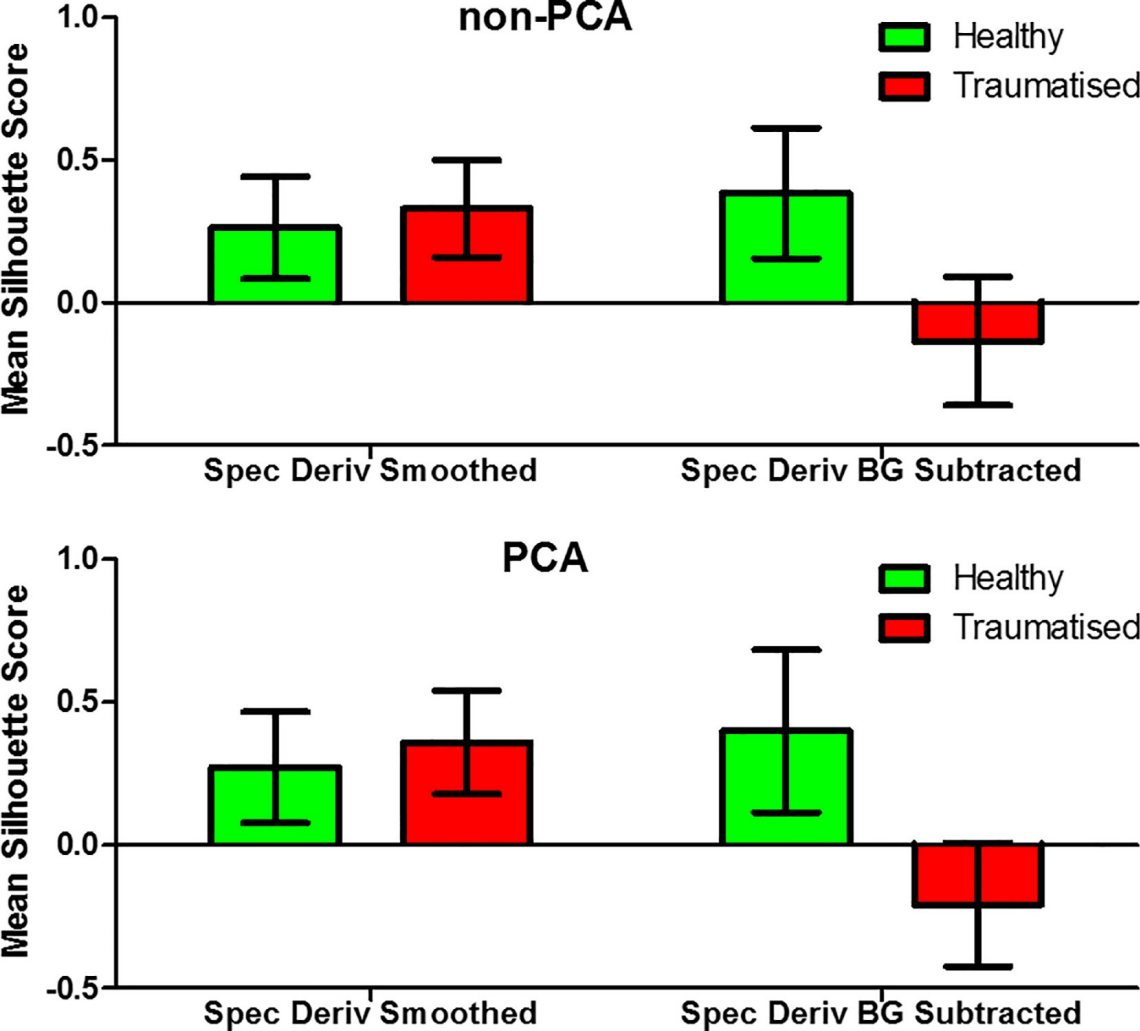
Silhouette cluster analysis. Mean data point silhouette score for each point within the healthy and traumatised cluster for (a) non-PCA and (b) PCA spectral derivate data types.

These results also highlight the methods ability to determine the state of the cellular health, independent of the trauma methods applied. Of the three different trauma methods used for this study, Triton X and H_2_O_2_, at the higher concentration of 5 mM, both induced necrosis of the cells. This form of cell death, which is not programmed and is unregulated, is different to apoptosis, or controlled cell death, which was induced with the lower H_2_O_2_ concentration of 100 μM. Despite these two different mechanisms, the imaging method and analysis can diagnose the healthy and traumatised samples. While the accuracy of the clustering algorithm through sensitivity and specificity calculations provides one metric for quantifiable comparison between data types, it is also possible to further assess the quality of each formed cluster. Silhouette cluster analysis is a method in which a data point is compared to every other within its cluster and a score from -1 to 1 is calculated for each point. A high score suggests a data point is closely matched with the other points within its own cluster, and a mean score can then be calculated for each cluster to quantify the quality of each formed cluster. With the sensitivity and specificity being the highest for both spectral derivative data types, the mean silhouette score for points within each cluster is shown in Fig 10(A) for the non-PCA and Fig 10(B) for the PCA results.

The positive mean silhouette score for both of the spectral (the non-PCA and PCA) of the healthy samples suggests the formation of good clusters for each of the different methods, with this also being observed in the spectral derivative smoothed data for the traumatised cluster. However, the data type with the highest sensitivity and specificity, >93% for both non-PCA and PCA, exhibits scores of <0 for the traumatised cluster, would could demonstrate that the formed clusters are either weak or artificial and that k-means clustering may not be the most suitable method as applied in this work. This may be due to the formation too few clusters and further work should investigate alternative clustering methods, such as hierarchical clustering, in order to determine the optimum number of clusters within each data set and confirm the reliability of the clustering algorithms [19, 43].

**Fig 11:**
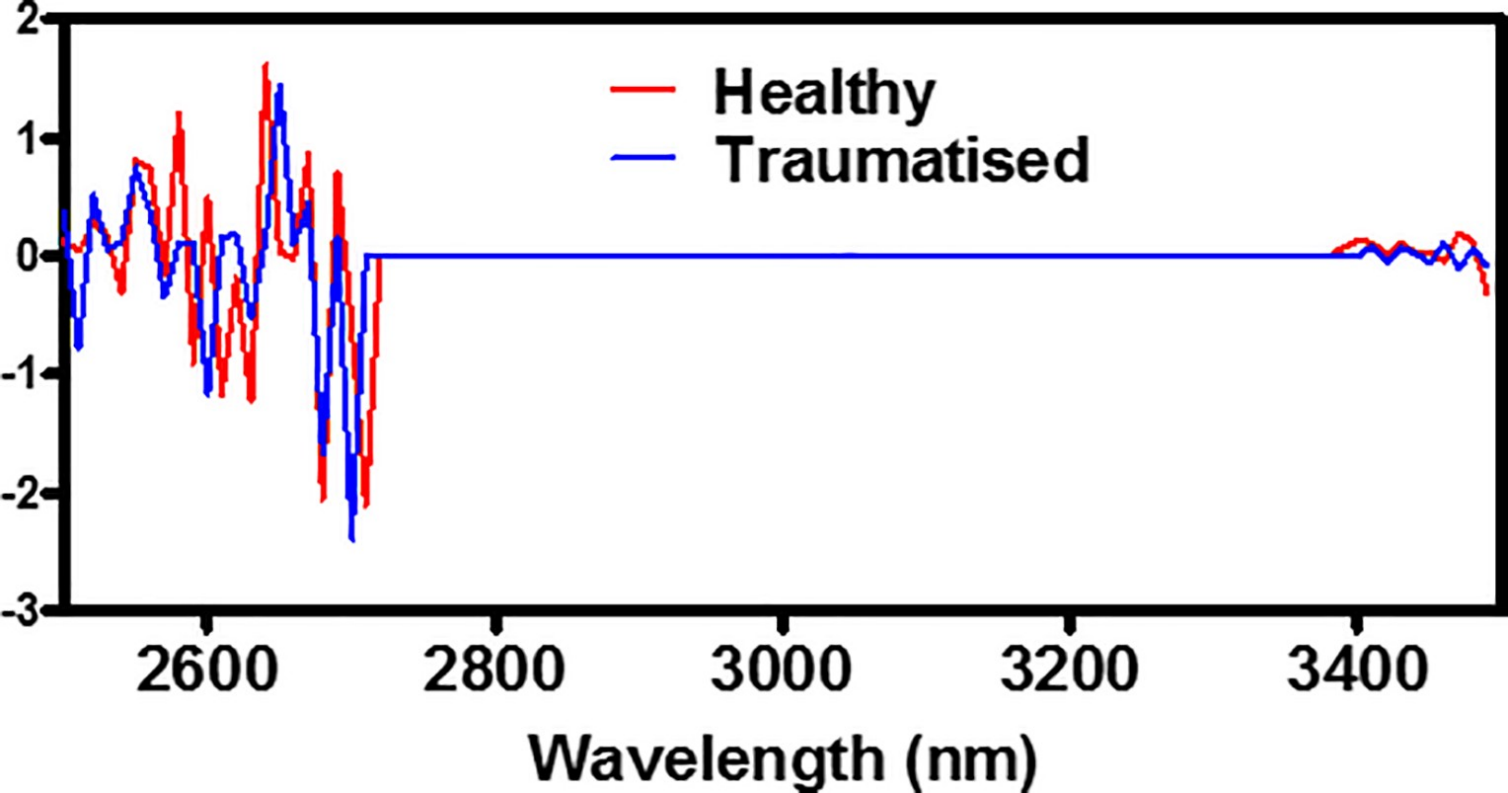
Healthy and traumatised derivate spectra. Comparison between a representative healthy and traumatised cell spectra of the spectral derivative background subtracted pre-processing data-type.

As seen in previous literature, the differences between both healthy and traumatised cells was detectable within the IR spectral region. The upper band of this study, 3000–3500 nm, has been investigated through the use of FTIR spectroscopy [12, 15], with differences attributed to the changes in cellular proteins and lipids during the apoptotic and necrotic processes. However, the sensitivity of a FTIR spectrometer is much greater than the NCI used here, and differences between the sample preparation must also be considered. Specialised sample preparation steps, including the drying and fixation of the cell monolayer have to be taken when using FTIR spectroscopy, which can affect the resultant spectra [13]. In this study, the cells were imaged within aqueous media and grown upon liquid-air interface inserts, allowing reflectance measurements to be taken without transmittance through the cell media solution. Although this provided a different method for imaging and spectral collection, differences between the healthy and traumatised samples were detectable, and accurate clustering of the two different cell health states was achieved. A comparison between a healthy and traumatised sample is shown in Fig 11, presenting the spectral derivative of the background subtracted data. This highlights a few key considerations and findings from this study. Firstly, as detailed above, the region between 3000–3500 has been considered with FTIR spectroscopy. For both the healthy and traumatised samples, this region is flat, due to the lower sensitivity of the NCI device, compared to that of an FTIR. However, a visual difference is observed from 3350–3500 nm, which has been shown to represent changes in lipid and protein conformational changes [12, 15]. Second, the 2500–2700 nm contains the largest proportion of the spectral derivative information over the entire imaging range. Previous studies have considered >3000 nm [15], along with limited investigation in the SWIR region incorporating 1000–2000 nm [16]. This study highlights a new region of investigation in live cell spectra, meriting further work within this spectral region. Finally, the differences between the two spectra shown in Fig 11 are subtle, which make them difficult to differentiate visually. The use of ML has shown the ability to separate these results with a high level of accuracy, correctly classifying >93% of the samples. The sensitivity and specificity can be combined to produce a single ‘accuracy’ metric for any given medical test. Due to the equal measurements of both healthy and traumatised samples, this metric is calculated by simply taking the mean of both values for each post-processing data-type. The resulting accuracy of both tests was above 95%, with the PCA before k-means observing the lower of the two tests at 95.5%, compared to the simpler k-means only accuracy of 96.1%. This study, conducted across the SWIR/MWIR spectral regions, draws a focus to investigating additional IR regions to aid in the diagnostics process. Current wound healing diagnostic procedures rely upon visual inspection by trained clinicians, which introduces increased subjectivity and varying results of accuracy [44]. The introduction of clinical imaging and ML diagnostic tools beyond the visible-NIR range will aid in this process, although further study into more complex cellular or tissue models will be required. Whilst this study has highlighted the existence of differences between healthy and traumatised cell culture samples, no information on the specific biological constituents that give rise to these differences has been shown. An increase in the number of samples tested, along with a thorough investigation into the biological changes that are occurring at the cellular level will further highlight the individual spectral contributions. A benefit of this presented method is to investigate the whole spectral range to identify any differences present, which is the only requirement for a diagnostic clustering purpose, with future work focussing upon each spectral contribution. Furthermore, a larger sample size would allow for the testing of further clustering and classification methods, with the aim of producing a binary classifier to clinically assess cellular health.

## Conclusion

In summary, the use of a SWIR/MWIR hyperspectral imaging device, along with machine learning algorithms, has been shown to differentiate and diagnose the spectral differences between healthy and traumatised cell cultures. Human dermal fibroblast cells were imaged using an NCI imaging device, with hyperspectral images collected between 2500–3500 nm with a 10 nm resolution. Spectral information for both healthy and traumatised samples, dosed with trauma inducing agents, were analysed using a variety of different pre- and post-processing methods. k-means clustering was applied to these different data-types, with the aim of correctly identifying the cellular health of each sample. The raw, background subtracted and smoothed data-types, although showing >85% for one of either sensitivity or specificity, were unable to accurately diagnose the cellular health of each sample. This accuracy was greatly improved to >95% for both the full data set and the dimensionally reduced set corresponding to the spectral derivative of the same background subtracted spectra, however the formed traumatised clusters were found to be weak (as compared to healthy) suggesting that k-means clustering as applied within this work may not be best suited to differentiate diseased cells from healthy cells. Although tested on a small number of samples, <100, the ability for simple pre- and post-processing machine learning algorithms demonstrates the future for clinical diagnostics of wounds and subsequent wound healing procedure. These methods are not applicable to wound relevant spectra, but for all spectral analysis problems where subtle spectral differences are undetectable using simple observation or classical techniques.
